# Associations Between Major Depressive Disorder, Multimorbidity Burden, and Inflammatory Biomarkers (NLR, PLR, MLR, SII, and MHR) in a Primary Care Population

**DOI:** 10.3390/jcm15114351

**Published:** 2026-06-04

**Authors:** Mehmet Yildiz, Ahmed Cihad Genç, Kubilay İşsever, Enes Zafer, Ali Muhtaroğlu, Merve Demir Yildiz, İrem Elgörmüş Zafer, Fevziye Türkoğlu Genç, Onur Öztürk

**Affiliations:** 1 Giresun Provincial Health Directorate Bulancak Sehit Er Enver Erdogan Family Health Center, Giresun 28300, Türkiye; 2 Private Clinic, İstanbul 34480, Türkiye; genccihad@gmail.com; 3 Department of Internal Medicine, Faculty of Medicine, Giresun University, Giresun 28200, Türkiye; kubilayissever@gmail.com; 4 Department of Internal Medicine, Sakarya Training and Research Hospital, Sakarya 54100, Türkiye; md.eneszafer@gmail.com; 5 General Surgery Clinic, Private Adatıp Hospital, Sakarya 54050, Türkiye; alimuhtarogluu@gmail.com; 6 Department of Child and Adolescent Psychiatry, Faculty of Medicine, Giresun University, Giresun 28200, Türkiye; mervedemiryildiz@gmail.com; 7 Department of Psychiatry, Faculty of Medicine, Sakarya University, Sakarya 54100, Türkiye; iremelgg@gmail.com; 8 Department of Internal Medicine, Kanuni Sultan Süleyman Training and Research Hospital, İstanbul 34303, Türkiye; fevziyeturkoglu@gmail.com; 9 Department of Family Medicine, Faculty of Medicine, Amasya University, Amasya 05100, Türkiye; onur.ozturk@amasya.edu.tr

**Keywords:** depression, inflammation, lipoproteins, HDL, major depressive disorder, primary health care

## Abstract

**Background/Objectives**: Major depressive disorder (MDD) frequently coexists with chronic diseases and inflammatory processes, particularly in primary care. The primary aim of this study was to investigate the association between MDD and multimorbidity burden (≥2 chronic diseases), while the secondary aim was to examine the relationship between MDD and routinely available inflammatory biomarkers. **Methods**: This retrospective cross-sectional study used electronic medical records from a Family Health Center in Türkiye between 1 January 2025 and 31 December 2025. Individuals aged ≥18 years with accessible records, complete laboratory data, and recorded sociodemographic/lifestyle variables were included. Patients were considered to have MDD if a psychiatrist-diagnosed MDD according to the Diagnostic and Statistical Manual of Mental Disorders, Fifth Edition (DSM-5), was recorded in the electronic medical system within the past year. Multimorbidity burden was defined as the total number of chronic diseases and was further dichotomized as ≥2 versus <2 for multivariable regression analysis. Inflammatory biomarkers were calculated from routine blood tests: neutrophil-to-lymphocyte ratio (NLR), platelet-to-lymphocyte ratio (PLR), monocyte-to-lymphocyte ratio (MLR), systemic immune–inflammation index (SII), and monocyte-to-HDL cholesterol ratio (MHR). Group comparisons and logistic regression were performed to evaluate associations with MDD. **Results**: A total of 3006 patients were analyzed; 375 (12.5%) had MDD. Participants with MDD were older and more frequently female, had higher body mass index and waist circumference, and exhibited a substantially higher burden of chronic comorbidities. PLR was higher in the MDD group (*p* = 0.019), whereas MHR was lower (*p* = 0.004); NLR, MLR, and SII did not differ significantly. In multivariable analysis, increasing age (OR 1.029, 95% CI 1.019–1.039, *p* < 0.001), female sex (OR 2.659, 95% CI 1.949–3.636, *p* < 0.001), and multimorbidity (OR 2.162, 95% CI 1.650–2.833, *p* < 0.001) were independently associated with MDD, whereas PLR and MHR were not independently associated after adjustment. **Conclusions**: In this large real-world primary care cohort, MDD was common and strongly linked to multimorbidity. Although PLR and MHR differed significantly between groups, they were not independently associated with MDD after adjustment, suggesting that these differences may be driven by multimorbidity and related clinical factors rather than independent disease-specific effects.

## 1. Introduction

Major depressive disorder (MDD) is a common chronic psychiatric condition that affects mood, motivation, behaviour and cognitive processes. It is associated with a high prevalence rate and substantial functional impairment [1,2]. According to current global estimates, depressive disorders affect around 280–350 million people worldwide, representing one of the leading causes of disability in all age groups [3]. The World Health Organization projects that depressive disorders will continue to contribute to the global burden of disease in the coming years, with MDD remaining one of the main causes of global functional disability [4].

Beyond its psychiatric manifestations, MDD is increasingly being conceptualised as a systemic condition associated with chronic medical diseases and metabolic disturbances [5]. The coexistence of MDD and chronic diseases is particularly common in primary care settings, where most patients with depression are first identified and managed [6]. Multimorbidity, defined as having two or more chronic diseases, has been consistently linked to higher rates of MDD and poorer clinical outcomes. The relationship between MDD and multimorbidity is bidirectional: chronic diseases increase the risk of MDD, while MDD adversely affects disease control, treatment adherence, and overall prognosis [7,8]. However, the biological mechanisms underlying this association remain incompletely understood.

Accumulating evidence suggests that chronic low-grade inflammation plays a central role in the pathophysiology of MDD through immune activation, cytokine dysregulation, and neuroinflammatory pathways [5]. This inflammatory hypothesis of MDD has led to growing interest in easily accessible hematological markers reflecting systemic immune activation.

Inflammatory indices derived from routine complete blood count parameters such as the neutrophil-to-lymphocyte ratio, (NLR) platelet-to-lymphocyte ratio (PLR), monocyte-to-lymphocyte ratio (MLR), and systemic immune–inflammation index (SII) are increasingly used as surrogate markers of systemic inflammation [9,10,11,12,13,14]. SII, calculated as platelet × neutrophil/lymphocyte, integrates three immune cell lines and is considered a more comprehensive marker of inflammatory status [9].

Similarly, the monocyte-to-high-density lipoprotein cholesterol (HDL-C) ratio (MHR) has emerged as a novel cardiometabolic inflammatory marker. Monocytes contribute to endothelial dysfunction and atherogenesis, whereas HDL-C exerts anti-inflammatory and antioxidant effects. Thus, MHR reflects the balance between pro-inflammatory and anti-inflammatory mechanisms [15]. Elevated MHR has been associated with cardiovascular disease, metabolic syndrome, and adverse clinical outcomes [16].

These indices have been extensively investigated in cardiovascular diseases, malignancies, metabolic disorders, and autoimmune conditions [9,12]. More recently, emerging evidence suggests that altered PLR, MLR, SII, and MHR values may also be observed in patients with depressive disorders, supporting the role of immune dysregulation in MDD [17]. However, findings remain inconsistent, and most studies have been conducted in psychiatric samples without adequately accounting for multimorbidity burden.

Given the close interplay between depression, chronic diseases, and systemic inflammation, evaluating these inflammatory biomarkers within a real-world primary care population may provide further insight into the biological underpinnings of MDD. Therefore, this study aimed to investigate the associations between MDD, multimorbidity burden, and inflammatory biomarkers -including PLR, MLR, SII, and MHR- in a cross-sectional primary care cohort.

## 2. Materials and Methods

### 2.1. Study Design, Setting, and Participants

This quantitative, retrospective study was conducted using anonymized electronic medical records of patients who visited the Giresun Provincial Health Directorate, Bulancak Sehit Er Enver Erdogan Family Health Center (FHC) in a primary care center in Türkiye between 1 January 2025, and 31 December 2025. All data were obtained by retrospectively reviewing data already recorded during routine clinical practice; no additional tests, interventions, or direct patient contact were involved. The target population consisted of adults of whom a total of 3006 patients were included in the study.

Inclusion criteria were: age ≥ 18 years; to apply to the FHC within the specified study period; availability of complete laboratory data allowing calculation of inflammatory biomarkers; accessible medical health records; and recorded sociodemographic and lifestyle variables.

In cases of multiple visits by the same individual during the study period, the most recent complete laboratory panel was selected. Each individual was included only once in the analysis to avoid duplication.

Exclusion criteria were: age < 18 years; the presence of acute or active conditions known to significantly influence systemic inflammatory markers, such as active viral or bacterial infection, acute inflammatory disease, chronic inflammatory or autoimmune disorders in an active phase, current antibiotic therapy, recent chemotherapy or radiotherapy, recent surgical intervention, or active malignancy; incomplete essential clinical or laboratory data; and patients whose electronic medical records were inaccessible or restricted.

A complete-case approach was used for analysis. Individuals with missing essential sociodemographic, clinical, or laboratory variables required for study analyses were excluded from the final dataset. No imputation procedures were performed because the missing variables involved key study parameters necessary for biomarker calculation and multivariable analyses.

### 2.2. Study Size

**Figure 1:**
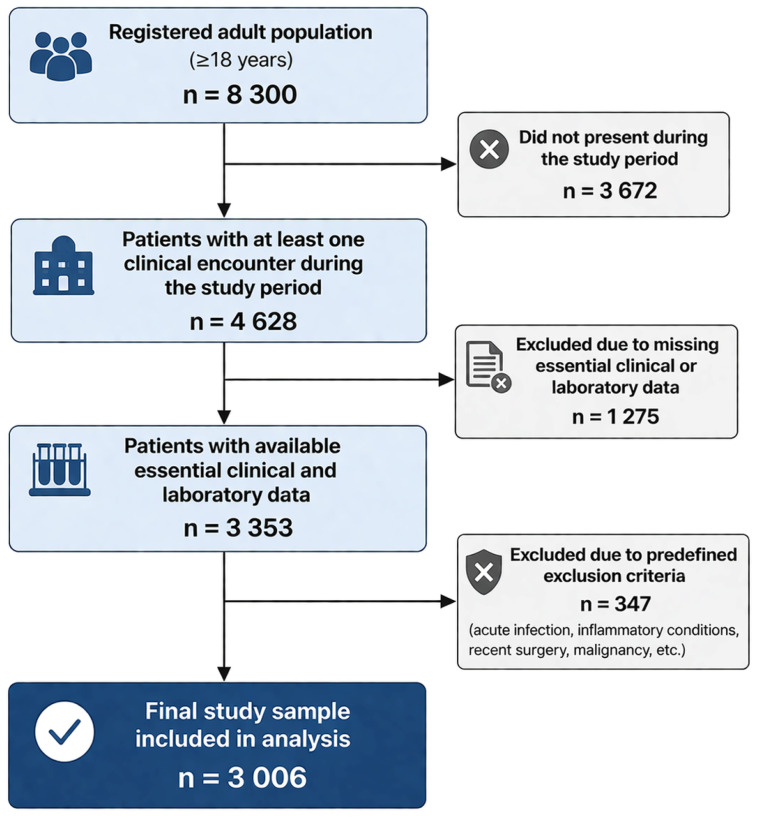
Flow diagram of participant selection from the registered population to the final analytical sample.

The source population of this study consisted of 8300 individuals aged 18 years and older who were registered with the relevant center. This FHC comprises four active family medicine units. All eligible patients meeting the inclusion criteria within the planned study period were included, resulting in a final sample of 3006 participants (Figure 1). As this was a retrospective study including all accessible and eligible individuals within the study timeframe, an a priori sample size calculation was not performed. This approach reflects a real-world data inclusion strategy rather than hypothesis-driven sampling.

Laboratory measurements were not obtained through a standardized screening protocol however reflected routine clinical practice. Therefore, available laboratory data primarily originated from individuals seeking healthcare services and undergoing physician-requested evaluations according to clinical needs. As a result, the final analytical sample may represent a subgroup with greater healthcare utilization, chronic disease burden, and clinical complexity rather than the entire registered primary care population.

### 2.3. Data Collection Tool

Data were retrospectively obtained from the following electronic medical systems:

- Family Medicine Information System (automation).
- Republic of Türkiye Ministry of Health e-Nabız system.
- Laboratory information system records.

No questionnaires were administered, no additional blood samples were collected, and no clinical interventions were performed. All data were anonymized and de-identified prior to analysis.

### 2.4. Variables

#### 2.4.1. Dependent Variable

The dependent variable was the presence of MDD (yes/no). Individuals who had been diagnosed with MDD by a psychiatrist according to Diagnostic and Statistical Manual of Mental Disorders, Fifth Edition (DSM-5) diagnostic criteria within the past year and whose diagnosis was recorded in the electronic medical system were classified as having MDD. This definition reflects a 12-month clinical diagnosis and does not necessarily indicate current episode activity at the time of data extraction. Therefore, individuals in remission may also have been included in the MDD group. In addition, detailed information regarding antidepressant treatment status, episode severity, chronicity, and remission status was not consistently available in the retrospective records. The proportion of MDD cases reflects the natural distribution of diagnosed depression in this real-world primary care population, rather than a pre-specified or matched sampling design.

#### 2.4.2. Main Independent Variables

Multimorbidity burden was defined as the total number of chronic diseases per individual and categorized as 0, 1, 2, 3, 4, 5, 6, 7, or 8 chronic diseases.

The following laboratory parameters were obtained from routine blood tests: HDL-C (mg/dL), lymphocyte (×10^9^/L), monocyte (×10^9^/L), neutrophil (×10^9^/L), and platelet (×10^9^/L).

Based on these parameters, the following inflammatory indices were calculated:

NLR: neutrophil/lymphocyte, MLR: monocyte/lymphocyte, PLR: platelet/lymphocyte, SII: (platelet × neutrophil)/lymphocyte, MHR: monocyte/HDL-C.

All indices were calculated using values obtained from the same laboratory assessment.

Serum HDL-C levels were measured using a Roche Cobas c501 autoanalyzer (Roche Diagnostics GmbH, Mannheim, Germany)^®^.

Complete blood count parameters (neutrophil, lymphocyte, monocyte, platelet) were analyzed using a SYSMEX XN-1000 fully automated hematology analyzer (Sysmex Corporation, Kobe, Japan)^®^.

#### 2.4.3. Covariates

Covariates included age (years), sex, marital status, current smoking, current alcohol use, body mass index (BMI) (kg/m^2^), waist circumference (cm), and regular physical activity. Regular physical activity was defined as engaging in moderate-intensity exercise at least three times per week.

The presence of chronic diseases was recorded based on electronic medical records and included diabetes mellitus, hypertension, hyperlipidemia, coronary artery disease, hypothyroidism, asthma and/or chronic obstructive pulmonary disease (COPD), cardiac arrhythmia, cerebrovascular disease, epilepsy, congestive heart failure, liver cirrhosis, chronic kidney disease, familial Mediterranean fever, and history of cancer.

### 2.5. Statistical Analysis

In the statistical analysis of the data, the distribution of continuous variables was assessed using skewness and kurtosis values, histogram plots, and the Shapiro–Wilk test. As most continuous variables did not demonstrate a normal distribution, they were expressed as median (25th–75th percentiles), and comparisons between two independent groups were performed using the Mann–Whitney U test. Normally distributed variables were expressed as mean ± standard deviation (mean ± SD) and compared using the independent samples *t*-test. Categorical variables were presented as number and percentage *n* (%). Chi-square tests were used to evaluate the relationships between two independent categorical variables. To evaluate the relationships between two groups, univariate analyses were performed first, and then multivariable analyses were applied to control for the effect of potential confounding variables. The selection of variables for the multivariable model was based on clinical relevance and potential confounding effects, rather than solely on univariate statistical significance. Multicollinearity diagnostics were assessed using variance inflation factors and tolerance values before multivariable modelling. Variables were selected according to study objectives and biological plausibility. PLR and MHR were included in the final multivariable model because they were the only inflammatory biomarkers demonstrating significant between-group differences in primary analyses and therefore represented the principal candidate biomarkers for adjusted analyses. NLR, MLR, and SII were not entered into the final model because they did not demonstrate statistically significant between-group differences in primary analyses and were not considered primary candidate variables for multivariable assessment. For inflammatory indices with small-scale values such as MHR and PLR, alternative rescaling or standardization approaches were considered primarily from an interpretability perspective, although no additional transformation beyond the predefined ratio calculations was ultimately applied. The results of the multivariate analyses were reported with regression coefficient (B), odds ratios (OR), and 95% confidence intervals (CI). The independent effects of the variables were evaluated. All statistical analyses were performed using the IBM SPSS Statistics version 26 software package, and a *p*-value less than 0.05 was considered statistically significant.

Use of Generative Artificial Intelligence: During manuscript preparation, ChatGPT (OpenAI, GPT-5.5) was used solely for the generation and refinement of Figure 1. No artificial intelligence tools were used for study design, data collection, statistical analysis, interpretation of results, or scientific decision-making. All outputs were critically reviewed and edited by the authors, who take full responsibility for the content of this manuscript.

## 3. Results

A total of 3006 patients were included in the analysis, of whom 375 (12.5%) had MDD.

### 3.1. Anthropometric and Lifestyle Characteristics

Participants with MDD were significantly older than those without MDD (57.11 ± 13.24 vs. 46.91 ± 16.70 years, *p* < 0.001). Female sex was more prevalent in the MDD group (72.27% vs. 49.71%, *p* < 0.001), and a higher proportion of participants with MDD were married (84.0% vs. 75.48%, *p* < 0.001). Current smoking and alcohol use were less frequent among individuals with MDD (25.6% vs. 34.66%, *p* < 0.001; and 3.47% vs. 8.59%, *p* = 0.001, respectively). Body mass index and waist circumference were significantly higher in the MDD group (30.76 ± 5.40 vs. 28.81 ± 5.51 kg/m^2^, *p* < 0.001; and 101.58 ± 12.37 vs. 97.36 ± 14.58 cm, *p* < 0.001, respectively). Regular physical activity did not differ significantly between groups (*p* = 0.066) (Table 1).

### 3.2. Comorbid Diseases and Multimorbidity

All major chronic comorbidities, except liver cirrhosis and familial Mediterranean fever, were significantly more frequent in participants with MDD. The prevalence of diabetes mellitus (22.4% vs. 12.2%), hypertension (62.4% vs. 31.05%), hyperlipidemia (24.53% vs. 10.22%), coronary artery disease (17.07% vs. 8.36%), hypothyroidism (14.67% vs. 6.27%), asthma and/or COPD (22.13% vs. 11.48%), cardiac arrhythmia (6.93% vs. 2.39%), cerebrovascular disease (3.47% vs. 0.99%), epilepsy (3.47% vs. 0.76%), heart failure (2.13% vs. 0.34%), and cancer (3.73% vs. 0.87%) were all significantly higher in the MDD group (all *p* < 0.001). Chronic kidney disease was also more common in the MDD group (1.07% vs. 0.23%, *p* = 0.027) (Table 2).

Multimorbidity burden differed markedly between groups (*p* < 0.001). While 57.35% of individuals without MDD had no chronic diseases, only 23.2% of those with MDD were free of comorbidity. In contrast, having two or more chronic diseases was substantially more common among participants with MDD (Table 3).

In univariate logistic regression analyses, female sex (OR 2.64), diabetes mellitus (OR 2.08), hypertension (OR 3.69), hyperlipidemia (OR 2.85), coronary artery disease (OR 2.26), hypothyroidism (OR 2.57), asthma and/or COPD (OR 2.19), cardiac arrhythmia (OR 3.04), cerebrovascular disease (OR 3.60), epilepsy (OR 4.69), congestive heart failure (OR 6.35), and cancer (past) (OR 4.40) were all significantly associated with MDD (all *p* < 0.001). Chronic kidney disease was also associated with MDD (OR 4.72, 95% CI 1.33–16.79; *p* = 0.027). Current smoking (OR 0.65, *p* < 0.001) and current alcohol use (OR 0.38, *p* = 0.001) were inversely associated with MDD (Table 4).

### 3.3. Inflammatory and Nutritional Parameters

Compared with individuals without MDD, those with MDD had higher HDL-C levels (50.62 ± 12.87 vs. 48.29 ± 12.28 mg/dL, *p* = 0.001) and lower hemoglobin levels (13.27 ± 1.59 vs. 13.94 ± 1.80 g/dL, *p* < 0.001). In addition, lymphocyte counts were slightly lower in the MDD group (median [IQR]: 2.21 [1.78–2.65] vs. 2.26 [1.84–2.75] × 10^9^/L, *p* = 0.024). No significant differences were observed in monocyte (0.53 ± 0.20 vs. 0.55 ± 0.18 × 10^9^/L, *p* = 0.090), neutrophil (3.90 ± 1.70 vs. 4.03 ± 1.63 × 10^9^/L, *p* = 0.129), or platelet (262.85 ± 68.83 vs. 259.17 ± 68.98 × 10^9^/L, *p* = 0.333) (Table 5).

Among inflammatory indices, PLR was significantly higher in patients with MDD (126.40 ± 47.96 vs. 120.01 ± 49.74, *p* = 0.019), whereas MHR was significantly lower (0.0113 ± 0.0056 vs. 0.0122 ± 0.0056, *p* = 0.004). NLR, MLR, and SII did not differ significantly between groups (*p* = 0.793, *p* = 0.903 and *p* = 0.803, respectively) (Table 5).

### 3.4. Multivariable Analysis

In the multivariable logistic regression model, increasing age (OR 1.029, 95% CI 1.019–1.039, *p* < 0.001), female sex (OR 2.659, 95% CI 1.949–3.636, *p* < 0.001), and the presence of multimorbidity (≥2 chronic diseases) (OR 2.162, 95% CI 1.650–2.833, *p* < 0.001) were independently associated with MDD. In contrast, BMI (OR 1.001, 95% CI 0.961–1.042, *p* = 0.976), waist circumference (OR 1.006, 95% CI 0.990–1.024, *p* = 0.454), current smoking (OR 1.264, 95% CI 0.944–1.692, *p* = 0.116), current alcohol use (OR 0.768, 95% CI 0.409–1.444, *p* = 0.412), and married (vs. single) (OR 1.283, 95% CI 0.918–1.794, *p* = 0.145) were not independently associated with MDD. Regarding the primary inflammatory indices, neither PLR (OR 1.002, 95% CI 0.999–1.004, *p* = 0.167) nor MHR (OR 1.060, 95% CI 0.801–1.460, *p* = 0.908) showed an independent association with MDD in the fully adjusted model (Table 6).

## 4. Discussion

This study provides real-world primary care evidence on the interplay between MDD, multimorbidity burden, and inflammatory biomarkers (including NLR, PLR, MLR, SII, and MHR). One of the principal strengths of this study is that it was conducted in a large, real-world primary care setting and enabled the evaluation of multimorbidity burden alongside laboratory-based indices within routine clinical practice. In this broad, real-world primary care sample, consistent with the literature, 12.5% of the study population (375 of 3006 patients) had a diagnosis of MDD [18]. This prevalence is noteworthy, as it reflects the substantial burden of depressive disorders encountered in routine primary care and underscores the clinical importance of systematically assessing both psychiatric and physical comorbidities in this setting. Compared with individuals without MDD, those with MDD exhibited significantly higher rates of diabetes mellitus, hypertension, hyperlipidemia, coronary artery disease, hypothyroidism, asthma and/or COPD, cardiac arrhythmia, cerebrovascular disease, epilepsy, congestive heart failure, chronic kidney disease, and a history of cancer. Participants with MDD also demonstrated a markedly greater overall chronic comorbidity burden and were significantly associated with inflammatory indices such as the higher PLR and lower MHR. Overall, individuals with MDD were older and more frequently female, and multivariable logistic regression analysis identified age, female sex and ≥2 comorbid diseases as independent factors associated with MDD.

Beyond comorbidity clustering, our findings suggest that certain low-cost, peripheral blood-based indices -particularly higher PLR and lower MHR- differed between groups in unadjusted analyses. However, these associations did not persist after multivariable adjustment, suggesting that they are unlikely to represent independent markers of MDD in this population. Accordingly, these findings should be interpreted as exploratory. In contrast, several other indices (NLR, MLR, SII) did not differ meaningfully between groups. Although smoking and alcohol use were inversely associated with MDD in unadjusted analyses, these associations did not remain significant after adjustment. These findings may reflect confounding by demographic and clinical factors and should be interpreted with caution.

Our results are consistent with large-scale evidence demonstrating that, in primary care datasets, MDD is associated with a broad spectrum of chronic diseases and higher multimorbidity counts. In a primary care analysis conducted in Scotland including more than 1.7 million patients, MDD was found to be associated with higher odds for each assessed physical condition and demonstrated a graded increase in the likelihood of having multiple coexisting disorders [19]. The findings identified in our study -namely that MDD was associated with chronic diseases (e.g., diabetes mellitus, hypertension, coronary artery disease) and with increasing multimorbidity burden, and was independently associated with having ≥2 chronic diseases- are consistent with the existing literature. From a clinical perspective, this suggests that in primary care settings, MDD should not be conceptualized as an isolated psychiatric diagnosis, but rather as a condition embedded within complex chronic diseases profiles, requiring the concurrent management of MDD and other comorbid diseases. However, it should be noted that the role of multimorbidity in the relationship between inflammation and MDD is conceptually complex. Multimorbidity may act as a confounder because chronic diseases are strongly associated with both systemic inflammation and MDD. At the same time, multimorbidity may also lie on the causal pathway between long-term inflammatory burden and depressive outcomes, or may represent an intermediate clinical state through which inflammation, cardiometabolic dysfunction, and psychological distress interact. Therefore, adjustment for multimorbidity may reduce confounding, but it may also attenuate biomarker associations through partial overadjustment if multimorbidity functions as a mediator rather than a purely external confounder. For this reason, the lack of independent associations between inflammatory indices and MDD after adjustment should not be interpreted as definitive evidence against biological links between inflammation and MDD, but rather as an indication that these associations are strongly embedded within the broader clinical context of chronic disease burden. Future longitudinal studies using prespecified causal frameworks are needed to distinguish confounding, mediation, and overadjustment more clearly. Although multimorbidity was operationalized using the commonly accepted definition of ≥2 chronic diseases, dichotomization may reduce information and potentially obscure dose–response relationships across increasing disease burden. Future studies may benefit from evaluating multimorbidity using continuous or ordinal approaches to better characterize gradients in chronic disease burden and their relationship with MDD. Beyond diagnostic categorization, MDD is frequently associated with substantial functional impairment and reduced quality of life. The burden of depressive symptoms may adversely affect social functioning, occupational performance, and overall well-being. In primary care populations, increasing multimorbidity burden may further amplify these effects and contribute to more complex clinical presentations.

In addition, the operational definition of MDD used in the present study may have introduced clinical heterogeneity within the MDD group. A psychiatrist-documented diagnosis within the previous 12 months does not necessarily reflect current episode activity at the time of data extraction and may include individuals with active depressive episodes, remission states, recurrent depression, or chronic depressive conditions. These clinical states may differ substantially in symptom burden, biological characteristics, and inflammatory profiles. Therefore, variability in disease stage and clinical course may have attenuated biomarker associations and contributed to the heterogeneous inflammatory findings observed across individuals with MDD.

According to the results of our study, PLR levels were statistically significantly higher in the MDD group (Table 5); however, in multivariable analysis, this association did not remain significant after adjustment for factors such as age, sex, and multimorbidity (≥2) (Table 6). When examining the literature on the association between MDD and the PLR, studies have reported heterogeneous and sometimes conflicting findings. A systematic review that compiled studies investigating the association between PLR and the prevalence of MDD in the literature up to April 2025 reported, a positive association between PLR and MDD [20]. Similarly, according to a meta-analysis that screened similar databases and included 18 studies, PLR values in 2264 patients with MDD were significantly higher than those in a control group comprising 2415 individuals [21]. In another case–control study including 59 healthy controls and 50 drug-free patients with MDD, PLR levels were reported to differ significantly in patients with MDD compared with the healthy control group [22]. These conflicting findings in the literature that contradict our findings may stem from differences in the number of patients included in the studies and in the study design.

However, there are also reports in the literature that do not support this association. For example, in a retrospective cross-sectional study conducted in a hospital in Beijing between January and December 2019, including 239 patients followed with a diagnosis of MDD and 241 controls, no significant association was identified between PLR and MDD [23]. In another meta-analysis evaluating the relationship between MDD and PLR, including 1393 cases and 1370 controls, no significant difference in PLR values was found between subjects with MDD and controls, and the authors emphasized the need for larger-scale studies [24].

When considered alongside the mixed findings in the literature, our results provide a nuanced view of the relationship between PLR and MDD. This study contributes to the limited body of evidence examining inflammatory indices in the context of multimorbidity in a real-world primary care population. Although PLR was significantly associated with MDD in unadjusted analyses, this association did not remain significant after adjustment for age, sex, and multimorbidity burden, suggesting that the observed association may be influenced by demographic and comorbidity-related factors rather than representing an independent biomarker of MDD. Therefore, PLR should be interpreted cautiously and not considered a standalone diagnostic marker. Prospective studies are needed to further clarify this relationship.

According to the results of our study, MHR levels were statistically significantly lower in the MDD group (Table 5); however, in multivariable analysis, this association did not remain significant after adjustment for factors such as age, sex, and multimorbidity. Although studies examining the relationship between MHR and MDD are limited, a study conducted in Türkiye by Öztürk et al. (2023) reported findings consistent with ours, demonstrating higher HDL-C levels in the MDD group compared with healthy controls, while no significant difference in monocyte levels was observed between the groups [25]. In contrast, a study in the literature that conflicts with our findings was conducted by Uyar and Ateş Budak (2022), who performed a retrospective case–control study including 120 patients with MDD and 124 healthy controls and reported that MHR was significantly higher in patients with MDD compared with healthy controls [26]. The discrepancy between our findings and those reported in the literature may be attributable, at least in part, to differences in study design, sample size, and population characteristics. Overall, these findings suggest that MHR is not consistently associated with MDD and should be interpreted cautiously. Further large-scale prospective studies are needed to better clarify this relationship. Although PLR and MHR demonstrated statistically significant differences in unadjusted analyses, the absolute differences observed between groups were relatively modest. Therefore, the clinical relevance of these findings should be interpreted cautiously, as statistical significance does not necessarily imply clinically meaningful discrimination at the individual patient level. The attenuation of these associations after multivariable adjustment further supports the interpretation that these biomarkers may have limited independent clinical utility and are likely influenced by broader clinical factors, including multimorbidity burden. In addition, the large sample size may have increased the likelihood of detecting statistically significant differences in uncertain clinical relevance.

In our sample, no significant between-group differences were observed in certain inflammatory indices (NLR, MLR, and SII) (Table 5). Findings from the literature are heterogeneous, with both positive and null associations reported. For example, a meta-analysis conducted by Su et al. (2022) to evaluate the relationship between MDD and NLR and MLR, 2580 MDD cases and 2664 controls were analyzed for NLR, and 744 MDD cases and 765 controls for MLR [24]. While no significant difference was found in MLR values between individuals with MDD and controls, NLR levels were reported to be significantly higher in the MDD group [24]. Similarly, Cheng et al. (2022) reported higher NLR values but no significant differences in MLR in a meta-analysis of 18 studies [21]. In contrast, individual studies have yielded varying results; for instance, Marazziti et al. (2022) reported higher levels of both NLR and MLR in patients with MDD [27], whereas Zhu et al. (2023) found no significant association between NLR and MDD; however, significant differences were observed in MLR and SII values [23]. NHANES-based studies, by Zheng et al. (2024) and Li et al. (2023) reported a significant positive associations between the SII and MDD [28,29] while a comprehensive meta-analysis conducted by Jiang et al. (2025) indicated that both NLR and SII levels were significantly higher in patients with MDD compared with controls [30]. Taken together, these findings indicate that the relationship between inflammatory indices (including NLR, SII, MLR) and MDD is complex and inconsistent across studies. The absence of significant differences in NLR, MLR, and SII in our sample suggests that MDD may not be characterized by a universal and uniform inflammatory profile; rather, inflammatory markers may gain relevance in specific biological subtypes or particular clinical contexts. These findings suggest the potential value of evaluating MDD within the framework of an “inflammatory phenotype” and highlight that real-world primary care data may yield results that differ from those obtained in more selected clinical samples. Beyond the routinely available inflammatory indices evaluated in the present study, emerging areas such as complementary interventions, postoperative psychiatric outcomes, neurodegenerative biomarkers, and novel molecular inflammatory pathways may further contribute to understanding the complex biological and clinical dimensions of MDD and warrant future investigation.

*Limitations*

Several limitations should be acknowledged. First, the cross-sectional design precludes causal inference, and the observed associations may reflect bidirectional or confounded relationships. Second, MDD is a heterogeneous condition, and analyses based on aggregate inflammatory indices may obscure biologically relevant subgroups. Third, the operational definition of MDD based on electronic medical records reflects a psychiatrist-documented diagnosis within the past 12 months and does not capture episode status, severity, chronicity, or remission; moreover, individuals without recent psychiatric evaluation may have been misclassified. In addition, the timing of psychiatric diagnosis relative to laboratory assessment was not standardized, and information regarding active psychiatric follow-up at the time of blood sampling was unavailable. Therefore, subgroup analyses according to active depressive episodes, remission states, recurrent depression, or chronic depressive conditions could not be performed. These factors may have contributed to clinical heterogeneity and may have attenuated or diluted associations between inflammatory biomarkers and MDD. Fourth, the study population represents a care-seeking group with available laboratory data, which may introduce selection bias. Individuals undergoing laboratory testing may differ systematically from the broader registered population regarding healthcare utilization patterns, chronic disease burden, symptom severity, and health-seeking behaviors. Therefore, participants included in the present analysis may represent a subgroup with greater healthcare engagement and clinical complexity, potentially influencing the observed associations. Because 1275 individuals were excluded due to missing essential clinical or laboratory data, the final analytical sample may also differ systematically from excluded participants, and selection bias cannot be excluded. In addition, the control group was not systematically screened for depressive symptoms, raising the possibility of undiagnosed cases. Furthermore, the mean age of the study population was relatively high for a primary psychiatric care sample. This characteristic likely reflects the real-world structure of the included primary care population, in which laboratory testing and chronic disease monitoring are more frequently performed among older adults and individuals with greater healthcare utilization. Therefore, the findings may more strongly reflect associations observed in middle-aged and older primary care populations rather than younger community-based populations. Fifth, detailed information on medication use was not available for analysis; in particular, antidepressant use was highly prevalent in the MDD group and nearly absent in the non-MDD group, precluding its inclusion in the model and representing a major source of potential confounding. Furthermore, antidepressants, antipsychotics, mood stabilizers, lipid-lowering agents, anti-inflammatory medications, antihypertensive drugs, antidiabetic therapies, and other treatments may influence inflammatory pathways, immune responses, HDL-C levels, monocyte counts, and cardiometabolic status. This issue may be particularly relevant for MHR, which directly incorporates HDL-C and may therefore be susceptible to treatment-related influences. Consequently, some observed biomarker differences may partly reflect treatment-related or comorbidity-related effects rather than MDD-specific biological mechanisms. Sixth, given the number of comparisons performed, there is a potential risk of type I error, and the findings should therefore be interpreted with caution. Finally, although the study reflects a large real-world primary care population, healthcare systems may differ substantially across countries regarding psychiatric referral pathways, access to mental health services, and chronic disease management models. Such differences may influence patterns of multimorbidity, healthcare utilization, and the observed relationships between MDD and inflammatory markers. Therefore, caution is warranted when extrapolating these findings beyond the Turkish primary care setting, and confirmation in prospective multi-center studies is warranted.

## 5. Conclusions

In this real-world primary care cohort, our findings suggest that, in primary care settings, MDD is strongly associated with common chronic diseases such as diabetes mellitus, hypertension, and coronary artery disease, as well as with a multimorbidity burden, which emerged as one of the most robust independent correlates of MDD in adjusted analyses.

Although PLR and MHR differed between groups in unadjusted analyses, these associations did not remain significant after adjustment and therefore do not appear to be independent of demographic characteristics, multimorbidity burden, and related clinical factors. Accordingly, these findings should be interpreted cautiously and should not be considered evidence of MDD-specific biomarker effects. Given the heterogeneous and inconsistent results in the literature, our findings suggest that these inflammatory indices may not be independent predictors of MDD in real-world primary care populations. Instead, it is likely that the observed associations are influenced by multiple disease and related clinical factors. Our findings may raise awareness regarding the integrated management of MDD and physical health conditions in Türkiye and worldwide. Future studies investigating the relationship between these inflammatory markers and MDD should incorporate multimorbidity as a potential confounding factor and include detailed medication data, while well-designed prospective studies are needed to further elucidate this association.

## Figures and Tables

**Table 1:** Anthropometric measurements and lifestyle characteristics according to major depressive disorder status.

Variable	With MDD (n = 375)	Without MDD (n = 2631)	Total (n = 3006)	*p* Value
Age (years), mean ± SD	57.11 ± 13.24	46.91 ± 16.7	48.28 ± 16.65	**<0.001**
Female sex	271 (72.27%)	1308 (49.71%)	1579 (52.53%)	**<0.001**
Married	315 (84.0%)	1986 (75.48%)	2301 (76.55%)	**<0.001**
Current smoker	96 (25.6%)	912 (34.66%)	1008 (33.53%)	**<0.001**
Current alcohol use	13 (3.47%)	226 (8.59%)	239 (7.95%)	**0.001**
Body mass index (kg/m^2^)	30.76 ± 5.40	28.81 ± 5.51	29.05 ± 5.53	**<0.001**
Waist circumference (cm)	101.58 ± 12.37	97.36 ± 14.58	97.89 ± 14.39	**<0.001**
Regular physical activity	12 (3.2%)	134 (5.09%)	146 (4.86%)	0.066

Footnotes: Values are presented as mean ± standard deviation or number (%), as appropriate. Comparisons between groups were performed using the independent samples *t*-test for continuous variables and the chi-square test for categorical variables. Regular physical activity was defined as engaging in moderate-intensity exercise at least three times per week. *p* values < 0.05 were considered statistically significant. Bold *p*-values indicate statistically significant differences (*p* <0.05). Abbreviations: MDD: Major Depressive Disorder, SD: Standard Deviation.

**Table 2:** Comorbid diseases according to major depressive disorder status.

Comorbidities	With MDD (n = 375)	Without MDD (n = 2631)	Total (n = 3006)	*p* Value
Diabetes mellitus	84 (22.4%)	321 (12.2%)	405 (13.47%)	**<0.001**
Hypertension	234 (62.4%)	817 (31.05%)	1051 (34.96%)	**<0.001**
Hyperlipidemia	92 (24.53%)	269 (10.22%)	361 (12.01%)	**<0.001**
Coronary artery disease	64 (17.07%)	220 (8.36%)	284 (9.45%)	**<0.001**
Hypothyroidism	55 (14.67%)	165 (6.27%)	220 (7.32%)	**<0.001**
Asthma and/or COPD	83 (22.13%)	302 (11.48%)	385 (12.81%)	**<0.001**
Cardiac arrhythmia	26 (6.93%)	63 (2.39%)	89 (2.96%)	**<0.001**
Cerebrovascular disease	13 (3.47%)	26 (0.99%)	39 (1.3%)	**<0.001**
Epilepsy	13 (3.47%)	20 (0.76%)	33 (1.1%)	**<0.001**
Congestive heart failure	8 (2.13%)	9 (0.34%)	17 (0.57%)	**<0.001**
Liver cirrhosis	0 (0.0%)	1 (0.04%)	1 (0.03%)	0.875
Chronic kidney disease	4 (1.07%)	6 (0.23%)	10 (0.33%)	**0.027**
Familial Mediterranean Fever	1 (0.27%)	5 (0.19%)	6 (0.2%)	0.551
Cancer (past)	14 (3.73%)	23 (0.87%)	37 (1.23%)	**<0.001**

Footnotes: Values are presented as number (%). Comparisons between groups were performed using the chi-square tests. *p* values < 0.05 were considered statistically significant. Bold *p*-values indicate statistically significant differences (*p* < 0.05). Abbreviations: COPD: Chronic Obstructive Pulmonary Disease; MDD: Major Depressive Disorder.

**Table 3:** Multimorbidity burden according to major depressive disorder status.

Number of Chronic Diseases	With MDD (n = 375)	Without MDD (n = 2631)	Total (n = 3006)	*p* Value
0	87 (23.2%)	1509 (57.35%)	1596 (53.09%)	**<0.001**
1	95 (25.33%)	535 (20.33%)	630 (20.96%)	
2	79 (21.07%)	247 (9.39%)	326 (10.84%)	
3	52 (13.87%)	193 (7.34%)	245 (8.15%)	
4	39 (10.4%)	108 (4.1%)	147 (4.89%)	
5	17 (4.53%)	29 (1.1%)	46 (1.53%)	
6	2 (0.53%)	8 (0.3%)	10 (0.33%)	
7	3 (0.8%)	2 (0.08%)	5 (0.17%)	
8	1 (0.27%)	0 (0.0%)	1 (0.03%)	

Footnotes: Values are presented as number (%). Comparisons between groups were performed using the chi-square tests. *p* values < 0.05 were considered statistically significant. Bold *p*-values indicate statistically significant differences (*p* < 0.05). Abbreviations: MDD: Major Depressive Disorder.

**Table 4:** Univariate logistic regression analysis for factors associated with major depressive disorder.

Variable	OR (95% CI)	*p* Value
Female sex	2.64 (2.07–3.33)	<0.001
Married	1.71 (1.28–2.28)	<0.001
Current smoker	0.65 (0.51–0.83)	<0.001
Current alcohol use	0.38 (0.22–0.68)	0.001
Diabetes mellitus	2.08 (1.59–2.72)	<0.001
Hypertension	3.69 (2.94–4.61)	<0.001
Hyperlipidemia	2.85 (2.19–3.73)	<0.001
Coronary artery disease	2.26 (1.67–3.05)	<0.001
Hypothyroidism	2.57 (1.85–3.56)	<0.001
Asthma and/or COPD	2.19 (1.67–2.88)	<0.001
Cardiac arrhythmia	3.04 (1.90–4.86)	<0.001
Cerebrovascular disease	3.60 (1.83–7.07)	<0.001
Epilepsy	4.69 (2.31–9.51)	<0.001
Heart failure	6.35 (2.44–16.56)	<0.001
Chronic kidney disease	4.72 (1.33–16.79)	0.027
Cancer (past)	4.40 (2.24–8.62)	<0.001

Footnotes: Odds ratios (ORs) and 95% confidence intervals (CIs) were obtained from univariate logistic regression analyses with major depressive disorder as the dependent variable. Abbreviations: CI: Confidence interval; COPD: Chronic Obstructive Pulmonary Disease; OR: Odds ratio.

**Table 5:** Inflammatory and nutritional indices according to major depressive disorder status.

Laboratory Parameters and Inflammatory Indices	With MDD (n = 375)	Without MDD (n = 2631)	Total (n = 3006)	*p* Value
HDL cholesterol (mg/dL), mean ± SD	50.62 ± 12.87	48.29 ± 12.28	48.57 ± 12.38	**0.001**
Hemoglobin (g/dL), mean ± SD	13.27 ± 1.59	13.94 ± 1.80	13.86 ± 1.79	**<0.001**
Lymphocyte count (×10^9^/L), median (IQR)	2.21 (1.78–2.65)	2.26 (1.84–2.75)	2.25 (1.84–2.73)	**0.024**
Monocyte count (×10^9^/L), mean ± SD	0.53 ± 0.20	0.55 ± 0.18	0.55 ± 0.18	0.090
Neutrophil count (×10^9^/L), mean ± SD	3.90 ± 1.70	4.03 ± 1.63	4.02 ± 1.64	0.129
Platelet count (×10^9^/L), mean ± SD	262.85 ± 68.83	259.17 ± 68.98	259.63 ± 68.96	0.333
NLR, median (IQR)	1.64 (1.28–2.21)	1.66 (1.28–2.15)	1.65 (1.28–2.16)	0.793
PLR, mean ± SD	126.40 ± 47.96	120.01 ± 49.74	120.81 ± 49.56	**0.019**
MLR, median (IQR)	0.23 (0.18–0.29)	0.23 (0.18–0.28)	0.23 (0.18–0.28)	0.903
SII, median (IQR)	420.83 (301.78–571.36)	420.20 (304.32–581.50)	420.66 (304.06–579.84)	0.803
MHR, mean ± SD	0.0113 ± 0.0056	0.0122 ± 0.0056	0.0121 ± 0.0056	**0.004**

Footnotes: Non-parametric variables were compared using the Mann–Whitney U test and were expressed as median (25th–75th percentiles), whereas parametric variables were compared using the independent samples *t*-test and were presented as mean ± standard deviation. *p* values < 0.05 were considered statistically significant. Bold *p*-values indicate statistically significant differences (*p* < 0.05). Abbreviations: HDL: High-Density Lipoprotein; IQR: Interquartile Range (25th–75th percentiles); MDD: Major Depressive Disorder; MHR: monocyte-to-high-density lipoprotein ratio; MLR: monocyte-to-lymphocyte ratio; NLR: neutrophil-to-lymphocyte ratio; PLR: platelet-to-lymphocyte ratio; SD: standard deviation; SII: systemic immune–inflammation index.

**Table 6:** Multivariable logistic regression analysis for major depressive disorder.

Variable	B	OR (95% CI)	*p* Value
Age	0.029	1.029 (1.019–1.039)	**<0.001**
Female sex	0.979	2.659 (1.949–3.636)	**<0.001**
Multimorbidity (≥2)	0.771	2.162 (1.650–2.833)	**<0.001**
Body mass index (kg/m^2^)	0.001	1.001 (0.961–1.042)	0.976
Waist circumference	0.006	1.006 (0.990–1.024)	0.454
Current smoker	0.234	1.264 (0.944–1.692)	0.116
Current alcohol use	−0.264	0.768 (0.409–1.444)	0.412
Married (vs. single)	0.249	1.283 (0.918–1.794)	0.145
PLR	0.002	1.002 (0.999–1.004)	0.167
MHR	0.053	1.060 (0.801–1.460)	0.908

Footnotes: Bold *p*-values indicate statistically significant differences (*p* < 0.05). Abbreviations: CI: Confidence interval; MHR: monocyte-to-HDL cholesterol ratio; OR: Odds ratio; PLR: platelet-to-lymphocyte ratio.

## Data Availability

The data presented in this study are available upon reasonable request from the corresponding author. The data are not publicly available due to privacy and ethical re-strictions.

## Abbreviations

The following abbreviations are used in this manuscript:

BMI	Body mass index
COPD	Chronic obstructive pulmonary disease
FHC	Family Health Center
HDL-C	High-density lipoprotein cholesterol
MDD	Major Depressive Disorder
MHR	Monocyte-to-high-density lipoprotein cholesterol ratio
MLR	Monocyte-to-Lymphocyte ratio
NLR	Neutrophil-to-Lymphocyte ratio
PLR	Platelet-to-Lymphocyte ratio
SII	Systemic immune–inflammation index
